# Learning Experiences Abroad for Residents in National Training (LEARN):  Results From a Scottish Training Programme Director Survey

**DOI:** 10.7759/cureus.80624

**Published:** 2025-03-15

**Authors:** Dominic Waugh, Magnus Johnston, Andrew Hayburn

**Affiliations:** 1 Trauma and Orthopaedic Registrar, NHS Greater Glasgow and Clyde, Glasgow, GBR; 2 General Surgery Registrar, NHS Lothian, Edinburgh, GBR; 3 General Practice Registrar, NHS Education for Scotland, West Region, Glasgow, GBR

**Keywords:** international study leave, medical education, study leave, training programme, training programme director

## Abstract

Introduction

Resident doctors (RDs) in UK training programmes are contractually entitled to take study leave (SL) to pursue activity related to progression of training. In 2024, NHS Education for Scotland (NES) announced a temporary change to SL policy, indicating international attendance at educational events would no longer be supported. NES is also responsible for administration of the RD study budget (SB) in Scotland - one of the lowest in the UK. There is little data regarding Training Program Director (TPD) views on RD use of SL to pursue professional development (particularly internationally) and appropriateness of SB. Here, we present TPD views on international SL and SB with an aim to contribute to wider policy discussions.

Methods

A cross-sectional survey consisting of closed and open responses was developed by the authors to evaluate a range of TPD opinions related to international SL and current SB. The survey was published in Microsoft Office Forms. Initial pilot testing was carried out by the authors before survey review by a Scotland TPD for content validity. The authors conducted a final re-test before distribution via TPD email addresses available via public domain. All TPDs in Scotland were eligible to respond, which was confirmed via survey response. No incentive or prize was offered. Responses were anonymised before review. Response percentage and chi-square analysis were undertaken using Microsoft Excel. Qualitative analysis of free text comments was conducted with assistance from Google Gemini AI Software, with prompts to assist "thematic analysis" before review by the authors to identify trends in response.

Results

In total, responses were obtained from 16% of invited TPDs (N=26) across a variety of medical specialties. International SL had been approved by 65% (N=17) of TPDs in the last 12 months. International SL was actively encouraged by 77% (N=20). TPDs were significantly more likely to encourage international SL if they had approved international leave requests within the last 12 months. SB was not considered appropriate to cover mandatory training costs by 85% (N=22) of respondents. Personal costs to trainees were estimated to be at least £1000 per annum in 88.5% (N=23) of training programmes. Annual SB was felt to be adequate for trainees by 12% of respondents (N=3). TPDs indicated overall support for international SL with regard to international collaboration, networking, research opportunity and access to learning opportunity not available in the UK. Responses highlighted concern that removing international SL could reduce the quality of medical training in Scotland and affect recruitment and retention of medical trainees.

Conclusion

There is relative consensus from Scottish TPDs on the importance of international learning during medical training, highlighting benefits for professional development, collaboration and education. The majority of TPDs view SB provided to trainees by NES as inadequate. Opportunity for suitable use of international SL could benefit RDs in Scottish training programmes with appropriate TPD oversight. Insights gathered from these responses could help inform policies to enhance international engagement in medical training programs within Scotland and the UK. Further exploration of differences in TPD opinion between specialties could highlight potential benefits of a level of autonomy in decisions made around resident doctor study budget funding and learning opportunities.

## Introduction

While all resident doctors in training within the UK National Health Service (NHS) are contractually entitled to study leave, access to funding for study purposes through local trusts or deaneries reflects regional variability and accessibility [1]. Medical trainees often face multiple challenges when accessing study leave with several organisations undertaking work to explore the nature of these difficulties. The British Geriatric Society surveyed trainees on issues related to study leave which highlighted the difficulty in balancing both clinical commitments and personal life to attend conferences - demonstrating some of the numerous barriers to accessing study leave [2]. Another proposed barrier to accessing study leave is associated cost. Previous analysis of UK physician income noted that medical graduates are unlikely to ever fully repay the levels of debt incurred during undergraduate study, even before any costs for postgraduate training have been considered [3]. Medical trainees often bear large levels of personal expense through training with differing personal costs noted across different training programs. Surgeons appear to bear some of the highest debt incurred during specialty training with an average personal cost associated with mandatory completion of training requirements ranging from £20,000 - £26,000 [4]. There is increasing discussion around disparity in study budget between training regions experienced by UK resident doctors in training. An analysis of study budget by the British Orthopaedic Trainee Association has highlighted variation in reimbursements for costs related to accommodation, sustenance, conferences and courses, leading to a call to reduce financial burden on individuals as a “Top 5 Priority” for the national association [5]. With financial pressures so apparent, trainees may turn to internationally delivered courses to reduce costs. The Royal College of Psychiatrists has highlighted further specific concerns around access to study budgets disproportionately affecting international medical graduates (IMGs) and those working less than full time [6]. Despite these costs, doctors are required to participate in numerous essential and mandatory courses as part of their training, in addition to courses that are “recommended” for professional development. Further to financial costs, there can be a personal cost associated with using annual leave to access continuing professional development activities when study leave cannot be accessed. Medical trainees have reported a desire to attend in-person events, agreeing that if they had access to study leave, they would be more likely to attend meetings face-to-face [2]. Some resident doctors choose to undertake these courses internationally, although the exact rate of this is unknown, for a wide range of reasons. While the global coronavirus pandemic significantly reduced the ability of individuals to attend academic conferences, subsequent work has highlighted the role these play in academic development, delivering multiple scientific and societal impacts [7]. With conferences offering a unique variety of mediums to engage with colleagues, this has led to development of foundational models encouraging connection and collaboration [8]. Further research has focussed on international mobility as a driver of scientific advances and productivity, noting that scientists who participate in international research visits are more likely to engage in knowledge transfer both at home and abroad [9]. Training Programme Directors (TPDs) often encourage trainees to present research at home or abroad, in keeping with curriculum requirements. Out-of-programme experience or training periods are now recognised for resident doctors, facilitating resident doctors undertaking periods of training abroad subject to TPD approval. Doctors have reported many benefits to undertaking clinical work internationally, including collaboration with international colleagues, developing skills that benefit their care of patients and renewing enthusiasm or motivation for work [10]. Within the United Kingdom many health matters are devolved, including policy regarding study leave which are respectively overseen separately by Health Care Education for England (HEE), NHS Wales, Northern Ireland Medical and Dental Training Agency and NHS Education for Scotland (NES) [11-14]. There are often subtle variations in these policies with regard to international study leave. NHS Wales highlights travel expenses will be paid if “no suitable courses are available locally”, with reimbursement possible for “UK air fares” [12]. In contrast, HEE suggests that “discretionary” international study leave can be granted, provided other curriculum requirements are met, with “one international conference / meeting… every three years” [11]. HEE further suggests they will consider funding the “full cost of travel”, in direct contrast to policy in NHS Wales [11,12]. In 2024, NES temporarily suspended international study leave for resident doctors in training in Scotland. Concerns regarding cost were one of the main reasons cited by NES in relation to their temporary removal of financial support and study leave time for overseas events - a decision that attracted criticism from professional representation bodies [15,16]. This variance in policy has the potential to disproportionately restrict opportunities available to resident doctors depending on which nation they live and work in. Whilst this decision was later reversed and is still under debate with the British Medical Association, this does reflect the current difficulties in accessing funding and study leave for resident doctors during training, particularly in Scotland. Resident doctor applications for study leave are approved or rejected by TPDs who have overall responsibility for delivery of medical training programmes. TPDs are therefore responsible for interpretation and implementation of national study leave policy and associated funding on a local basis. There is evidence that personal TPD opinions can influence management of parental leave applications, with studies demonstrating that program directors in a number of specialties perceive parental leave to negatively affect resident research, clinical skills and dedication to patient care [17]. With variance in support among TPDs with regard to parental leave, it is plausible that TPDs may also share a variance in opinion towards international study leave, though there is little in the literature discussing this.

Inconsistency in leave policies has been noted to contribute to dissatisfaction among medical trainees, potentially creating conflict for trainees regarding balancing demands of professional and personal life [18]. While some TPDs have expressed concerns that time away from work could affect training and skill acquisition, performance evaluations have not supported these opinions [19]. Other studies have highlighted that TPDs that support leave policies can be beneficial for resident doctor wellness and academic pursuits with limited impact on clinical ability or career advancement. TPDs have reported that a lack of institutional support can lead to barriers around handling leave requests [20]. The majority of research evaluating TPD opinions toward leave policies originates from the US and focuses on parental leave for residents. In the US it has been reported that leave policies, particularly parental leave, may be inconsistently implemented across training programmes, leading to disparities in resident support [21]. The limited research in this area from the UK highlights difficulties in evaluating TPD opinions towards study leave policies when there is inconsistency in delivery across countries and regions [11-14]. Literature surrounding UK TPD viewpoints on leave as a whole is very minimal with study leave being even lesser. The authors note discussions with some Scottish TPD directors with regard to potential changes in international leave policy, with concern highlighted regarding the implications of removal of support for international study leave from their training programme. As this was purely anecdotal, the authors wished to evaluate if TPD opinions related to international study leave and study budget were in keeping with concerns raised by unions [15,16] and by trainees [4-6]. We aimed to document attitudes toward international study leave and whether policies reducing resident doctor access to this learning opportunity could affect training programme delivery. Additionally, we sought to develop areas for future research and policy development.

## Materials and methods

The Learning Experiences Abroad for Residents in National Training (LEARN) survey questions were created by the authors, with the aim to evaluate a wide range of opinions related to international study leave and collect a comprehensive overview of thoughts related to training opportunities outside of the UK and adequacy of trainee study budgets. A cross-sectional survey allowed for a rapid response "snap shot" of viewpoints on international study leave, at a time when there was rapid policy change within NHS Scotland. Questions comprised closed questions with participants required to select from the options presented and free text responses. Additional questions comprised Likert scales ranging from 1-5 with descriptors ranging from: "completely disagree", "somewhat disagree", "neutral", "somewhat agree" and "completely agree". Questions pertaining to international study leave were phrased both in support of and in opposition to study leave in an effort to minimise selection bias in the results. Initial pilot testing was carried out by the authors before survey review by a Scotland TPD for content validity. The authors conducted a final re-test before distribution. 

The survey was published in Microsoft Office Forms, a web-based tool within the Office Suite (Microsoft, Redmond, WA, USA), that allows users to create online surveys, quizzes, polls and forms for collecting data. The survey was sent electronically to all Scottish TPD email addresses available publically through NHS Scotland online domain and advertised through email communication via Microsoft Office Outlook. The survey was open for completion for a period of six weeks (1st October 2024 - 10th November 2024). While TPDs were encouraged to share their views via the survey there was no incentive or prize offered. A further reminder was posted to all TPD email addresses before the conclusion of the study period.

All TPDs in Scotland were eligible to complete the survey, being confirmed by answering "Yes" when asked on the survey if the respondent was a "Scottish Medical Training Programme Director". Some 171 TPDs were eligible to respond from online listed email addresses, though some returned as "invalid". Additionally, security protocols were activated on Microsoft Office Forms to allow only respondents with a verified NHS Scotland email address to respond once, with participants unable to submit duplicate entries. All responses were anonymised, with each repsonse given a numerical identifier. No institutional or ethical review board approval was required or sought for this survey. Participants were informed that their responses could be used in presentation or research. Data was collected digitally and stored securely in line with General Data Protection Regulations via NHS Outlook. Responses were summarised in graphical and tabular format by Microsoft Office Forms. Descriptive statistics and chi-square testing were conducted with Microsoft Excel. Qualitative analysis of free text comments was conducted with assistance from Google Gemini software. Prompts given to Gemini reflected the request prompt for "thematic analysis" of anonymised comments. The results were then reviewed by the authors to identify whether trends and differences in collected data were apparent. A full copy of the LEARN survey is included in Appendix 1 with additional question responses to Likert scale questions shown inthe Resultssection.

## Results

**Figure 1:**
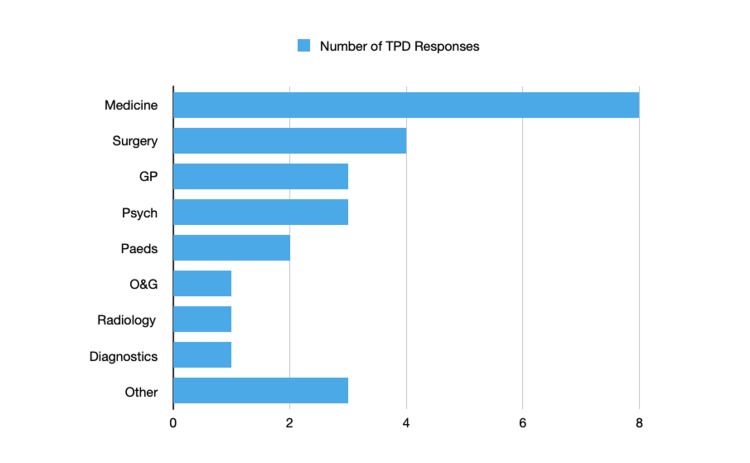
Number of Training Program Director (TPD) Responses by Specialty GP: General Practice, O&G: Obstetrics and Gynaecology

In total, N=26 responses from TPDs were obtained from a total of 165 operational email addresses, giving a response rate of 16%. Responses were received from TPDs responsible for multiple specialties including Diagnostic Specialties (N=1), General Practice (N=3), Medical Specialties (N=8), Obstetrics and Gynaecology (N=1), Paediatrics (N=2), Psychiatry (N=3), Surgical Specialties (N=4), Radiology (N=1) and Other (N=3). This is illustrated in Figure 1.

**Figure 2:**
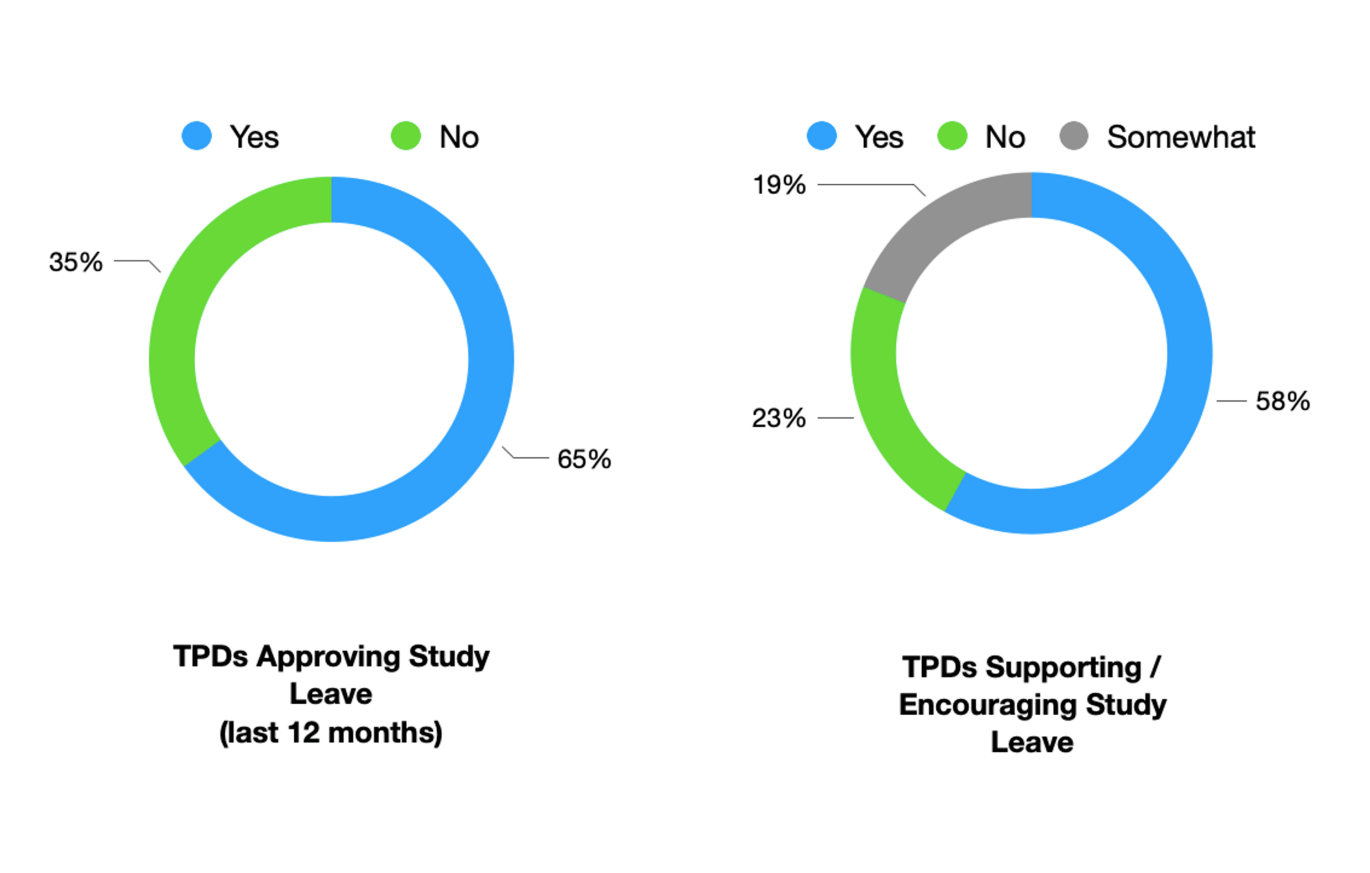
Training Program Director (TPD) Rates of Study Leave Approval and Supporting/Encouraging Leave

International study leave had been approved by 65% (N=17) of TPDs in the last 12 months and had not been approved in the last 12 months by 35% (N=9). The use of international study leave was “actively encouraged / supported" by 58% (N=15), “somewhat” encouraged by 19% (N=5) and not encouraged by 23% (N=6). When asked if international study leave was considered mandatory for training progression and attainment of a certificate of completion of training (CCT), 100% (N=26) of TPDs answered "no". This is summarised in Figure 2*. *A chi-square test of independence was performed to examine the relationship between support for international study leave and whether approval was given for trainees to attend leave in the last 12 months. The relationship between these variables was significant, X2 (2, N = 26) = 9.3577, p = <0.05.

**Figure 3:**
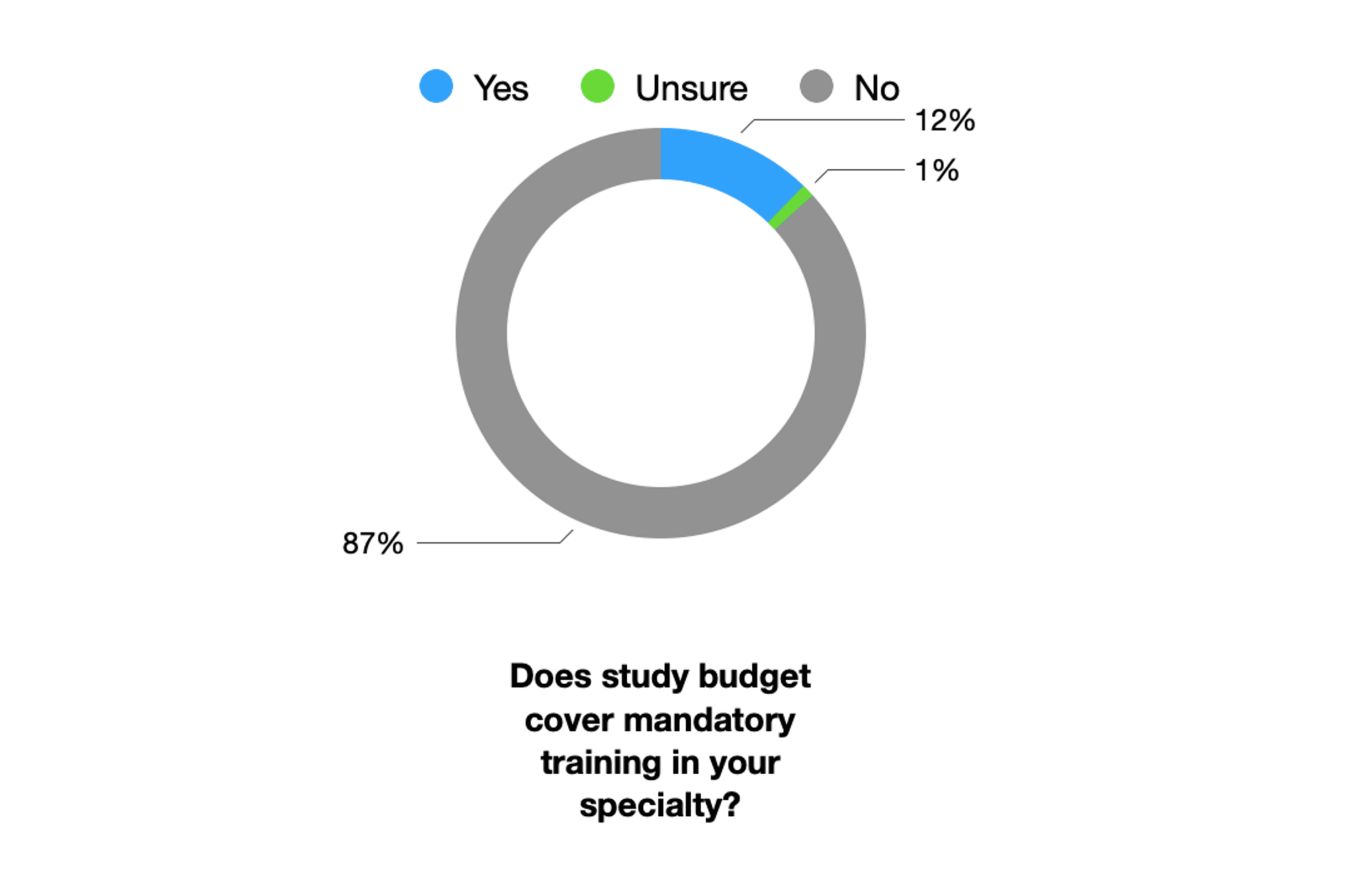
Training Program Director (TPD) Responses to Adequacy of Trainee Study Budget for Mandatory Training Costs

Resident doctor study budget in Scotland (£600 per annum at time of writing) was not considered appropriate to cover mandatory costs for progression in specialty training by 85% (N=22) of TPD respondents. Trainee study budget was considered appropriate for covering mandatory progression costs by 12% (N=3) of respondents; 4% (N=1) were unsure if study budget was appropriate. This is summarised in Figure 3*.* TPDs were asked if they felt the £600 annual NHS Scotland study budget was appropriate to support non-mandatory trainee needs and aspirations: 69% (N=18) responded "no" with 19% (N=5) responding “somewhat no”. TPDs felt this budget was appropriate in 12% (N=3).

TPDs were then asked to estimate if personal costs were incurred by trainees to cover any NHS Scotland study budget shortfall to attain mandatory training needs. Trainees were estimated to incur further personal costs by 88% (N=23) of TPDs. It was estimated by TPDs that trainees incurred mandatory costs ranging from up to £1000 per annum (68%, N=17) to £3000 (24%, N=6). A minority of TPDs (8%, N=2) estimated trainees did not incur additional personal expenditure annually to attain mandatory training requirements.

TPDs were asked whether they felt potential changes to NES policy to not support international study leave was good for their Training Programme. The majority (77%) responded “no” (N=15) or “somewhat no” (N=5). One respondent (N=4%) felt removing international study leave was good for their programme, with 19% of respondents (N=5) being “neutral”.

TPDs were asked to review several statements with regard to international study leave and select a response on a Likert scale ranging from "completely disagree" to "completely agree". Questions were phrased in support and in opposition of international study leave in order to reduce bias. Responses attained are expressed as percentages of total number of respondents (N=26) to one decimal place in Table 1.

**Table 1:** Training Program Director (TPD) Likert Scale Agreement Responses to Statements Pertaining to International Study Leave Figures are expressed as a percentage of total number of respondants (N=26) CCT: certificate of completion of training

Statement	Completely Disagree	Somewhat Disagree	Neutral	Somewhat Agree	Completely Agree
Trainees should collaborate with international peers			26.9%	23.1%	50%
Trainee research is better with international collaboration		3.8%	15.4%	50%	30.8%
Trainees learn less on international study leave	53.8%	15.4%	26.9%	3.8%	
International leave can reduce stress / burnout in trainees	11.5%	23.1%	42.3%	7.7%	3.75%
International study leave is not value for money	26.9%	23.1%	23.1%	26.9%	
Trainees can undertake funded international learning opportunity (industry, fellowship, scholarship etc)	3.8%	23.1%	26.9%	34.6%	11.5%
Trainees can undertaken international learning opportunity not available in the UK	8%	12%	12%	28%	40%
Removing international study leave will reduced the quality of medical training in Scotland	7.7%	3.8%	11.5%	23.1%	53.8%
International study leave can help trainees achieve CCT	11.5%	11.5%	30.8%	26.9%	19.2%
International study leave can help trainees secure a consultant job / posting post CCT	12%	12%	20%	36%	20%
Removing international study leave will have no effect on recruitment and retention of trainees in Scotland	46.2%	34.6%	7.7%	11.5%	

## Discussion

Overall trends in TPD responses

To our knowledge, this is the first project to explore UK TPD views regarding resident doctor use of international study leave. Additionally, this is the first published survey of Scottish TPD views regarding current rates of study budget funding available to NHS trainees in Scotland. The majority of TPDs actively supported international study leave to some degree (76%, N=20), with more than half (65%, N=17) approving international study leave in the last 12 months. Respondents who approved international leave in the past 12 months were significantly more likely to actively support international study leave in their training programme (p <0.05). Most TPDs (85%, N=22) responded that the NHS Scotland study budget (£600 per annum) was insufficient to cover mandatory training costs for progression and completion of training. Personal costs to trainees were estimated to be at least £1000 in 88.5% (N=23) of training programmes responding. The majority of respondents indicating study budgets are insufficient supports calls from trainee bodies to make funding a top priority to avoid personal costs to resident doctors and reduce personal financial burden [3-5]. International study leave was supported overall by 77% of TPDs (N=20). Several statements were presented with regard to international study leave and funding: the majority of TPDs indicated overall agreement. There was agreement that trainees should collaborate with international peers (73% agreement) and that research was better with international collaboration (81% agreement). It was felt that trainees did not learn less when taking international study leave (69% agreement) and that there was access to international learning opportunity not available anywhere in the UK (69% agreement). Responses suggested that removing international study leave would reduce the quality of medical training in Scotland (73% agreement) and could affect recruitment and retention of trainees in Scotland (81% agreement). This general consensus is in keeping with the perceived positive benefit of international study leave contributing to greater international social integration, collaboration and contributions to research [7,8]. Several statements presented had a greater spread of assent and dissent. TPDs were less convinced that international study leave reduced trainee stress/burnout (31.3% disagreement), represented value for money (50% disagreement) or could be accessed as a separately funded learning opportunity within their own specialty (31% agreement, 31% disagreement).

Qualitative analysis

Free text responses regarding the positive and negative aspects of TPD views on international study leave were qualitatively analysed with the aid of Google Gemini software. Several key themes were identified including the importance of international events for professional development, educational value and collaboration with colleagues. Concerns were raised regarding the removal of international study leave in relation to potential impacts on recruitment and practice in Scotland in comparison with the rest of the UK. It was also noted that there are challenges for TPDs in facilitating international study leave in relation to accessibility, funding and the need to balance service need with time out of training. These findings are summarised in Table 2*. *

**Table 2:** Free Text Analysis Regarding Training Program Director (TPD) Views on International Study Leave

Identified Theme	Free Text Analysis
Importance of International Events for Continuing Professional Development and Collaboration	Some respondents noted that participation in international events was beneficial for continuing professional development. This exposure to ‘best practice’ is seen as a means to acquire new skills and knowledge that can be brought back to the UK, ultimately benefiting patient care. Respondents also highlighted the value of international conferences for networking and collaboration. Many noted that these events provide opportunities to connect with peers, share research, and establish professional relationships that are vital for clinical practice. A potential loss of international cooperation is viewed as a serious concern, particularly by smaller specialties where such connections are crucial for obtaining specialist advice or second opinions.
Educational Value	The educational benefits of attending international meetings are frequently mentioned. Respondents point out that these events often cover topics not typically addressed in UK meetings, such as the effects of climate change on health and new emerging treatments. This broadens the educational experience for trainees and fosters a more comprehensive understanding of global health issues.
Impact on Recruitment and Practice	There are concerns that limiting international learning opportunities could impact the plans of future consultants and therefore negatively impact recruitment in Scotland, particularly compared to England. Respondents expressed that cost-cutting measures may have long-term implications for the quality of training and the attractiveness of Scotland as a place for medical professionals to work.
Specialty-Specific Insights & ‘Mixed’ Perceptions	Different specialties exhibit varying levels of engagement with international conferences. For instance, Urology and Infectious Diseases underscore the necessity of international guidelines and experiences, while some respondents from other specialism (such as General Practice) indicate less relevance. This suggests that the perceived value of international exposure may depend on the specific context of each specialty.
Concerns About Accessibility and Funding	Financial constraints associated with attending international meetings are highlighted. Respondents often highlighted that the study leave budget is inadequate, with specific concerns about the £600 allowance barely covering travel and attendance costs. This raises issues of accessibility and equity in opportunities for trainees and there are concerns this could particularly impact trainees in remote and rural areas and less-than-full-time trainees.
Balancing Needs of Trainees and Service	There is recognition of the challenges in accommodating lengthy periods out of program while balancing the needs of the trainee population as a whole when planning rotations. Time out of program for significant periods of study leave can negatively affect service provision and access to in-work training opportunities. This complexity underscores the need for careful management of international opportunities within the constraints of training programs.

Policy recommendations

Overall, this survey evidence demonstrates that there is relative consensus from TPDs on the importance of international exposure in medical training, highlighting benefits for professional development, collaboration, and education. However, significant challenges related to funding, accessibility, and management of international study leave opportunities are also evident. Addressing concerns around accessibility of international opportunities is important for ensuring equitable opportunities and ensuring that future consultants are well-equipped to meet the demands of their specialties. The insights gathered from these comments could inform policy decisions and strategies to enhance international engagement in medical training programs. It is important to note that not all TPDs are in support of international study leave, suggesting that this learning opportunity may not be as relevant in all training programmes. A difference in TPD opinion towards international leave policy is in keeping with previous surveys showing differences in support for parental leave policies [17-20]. Given the responses obtained within the limitations of this study, we would recommend that NES does not introduce significant national policy changes restricting access to international study leave without appropriate consultation with Scottish TPDs. Additional stakeholders and professional trade bodies should be invited to contribute to discussions so a mutually agreeable policy is developed.

Data gathered regarding TPD opinions related to overall study budget is of significant concern. Overall, respondents suggest that the study leave budget provided annually to trainees across NHS Scotland is felt to be inadequate by those running medical training programmes, with resident doctors taking on high levels of personal cost every year to continue in their job. This has significant implications for training future consultants. It is hoped that constructive discussion can be had nationally around how to optimise access to funding for trainees across Scotland to provide not only a "mandatory" standard of training, but to allow trainees access to training and education from international experts to deliver a world-class health service for Scotland's population. Despite this, we note that these results may not be entirely generalisable to all medical training programmes within NHS Scotland and it is important to consider specialty-specific needs. The authors would recommend future national NES study budget policies consider ensuring that resident doctors have mandatory training costs for their specific specialty training programme covered as a minimum requirement. Additionally, we would encourage local oversight of additional funding for recommended and aspirational specialty-specific courses where TPDs feel educational programmes and individuals could additionally benefit.

Future research recommendations

With national trainee surveys showing high rates of stress, burnout and mental health issues among medical trainees [22], the role of international study leave in improving staff wellbeing should be considered in future work. Similarly, it would be useful to compare TPD opinions on perceived quality of domestic and international training, educational and research opportunities to gain further insight into "value for money" and available funding streams for international study leave.

Study budget was felt to be adequate for only 12% of respondents (N=3), all of whom were TPDs in General Practice. In addition, TPDs (N=5) who neither supported international study leave nor approved any in the last 12 months were from Psychiatry and General Practice Training Programmes, suggesting the potential for differing needs between training programmes. It therefore is understandable that TPDs who do not support international study leave also view study budget as adequate for mandatory training that can be completed in the UK. There could be multiple contributing factors to this. For example, there is an increased number of mandatory courses required for hospital specialisms, such as Advanced Trauma Life Support and Advanced Life Support. Hospital specialism courses often have a "practical" component, with larger costs possibly explained by larger costs equipment costs or use of cadaveric laboratories. Whilst some literature exists indicating GPs in particular have reported benefits of international working [10], there may be differences in international health systems in family medicine and psychiatric practices which may contribute to TPDs in these areas viewing international exposure as less necessary. Further, UK GP and Psychiatry training is shorter than medical and surgical specialties training pathways, leaving trainees limited time to pass necessary professional exams and access study leave opportunity. It is possible that these exams are the priority for TPDs in these specialties and perhaps international collaboration becomes more relevant for consultants in these areas than trainees. Future research should conduct a specialty-stratified analysis to clarify specialty-specific opinions on study budget requirements and mandatory leave for trainees and consultants.

Limitations

Limitations of this study include the limited response rate (N=26, 16%) of all invited TPDs, with potential selection bias in participants. It could be the case that responses were primarily obtained by motivated strong supporters or detractors of international study leave and that those with less strong opinions did not respond. This "snap shot" survey was designed for rapid distribution and analysis at a time of rapid NES policy change in order to facilitate national discussions on the topic as early as possible, with the relatively short study period possibly contributing to a reduction in response rate. Additionally, opinions from several training programs were not collected despite invitation. As TPDs are a large heterogeneous group, insights into their specialty may not be apparent to those with less intimate knowledge of their specialism. Those TPDs not responding may have their own unique opinions not reflected in the responses to this survey. However, while the authors recognise that our responses fall below a truly representative sample size for this group, we believe the results are generally applicable as responses to other surveys of TPDs in the literature demonstrate similarly underpowered response rates [18-20]. These results cover a broad range of medical specialisms, suggesting generalisability. TPDs from across medicine and surgery are represented with many other areas of medical practice included, both patient-facing (psychiatry, obstetrics and gynaecology, paediatrics and general practice) and non-patient-facing (diagnostics, radiology and other) specialties. Improvements in response rate could be facilitated by measures such as opening a survey for longer periods, alternative methods of contact with TPDs and designing a follow-up survey with key stakeholders to encourage responses.

## Conclusions

UK TPD views are presented regarding the use of international study leave and study budget by resident doctors in training across Scotland. Resident doctors are perceived by TPDs to bear substantial personal costs to pursue training, with surveyed Scottish TPDs viewing the annual study budget as insufficient to cover mandatory costs.

TPDs are significantly more likely to promote the use of international study leave if they have approved requests within the last 12 months. Overall, there is consensus on the importance of international exposure in medical training, suggesting TPD support for the appropriate use of international study leave to bring benefits to their training programme. Concerns were raised by TPDs around the retention of current trainees and attractiveness of Scottish training programmes should international study leave and funding be revoked. Further research is required to inform policy decisions around optimisation of study leave and budgets, both in comparison to other UK nations and between specialties. The authors recommend that relevant stakeholders be engaged in future policy decisions.

## Appendices

Appendix 1: LEARN Survey

**Table 3:** Learning Experiences Abroad for Residents in National Training (LEARN) Survey

Question	Available Responses								
Are you a Scottish Medical Training Programme Director?	Yes	No							
What training programme are you responsible for?	General Practice	Medical Specialty Practice /(Cardio, Resp, Gastro etc)	Paediatrics	Psychiatry	Radiology	Obstetrics & Gynaecology	Surgical Specialty (General, T&O, Vascular etc)	Other	Diagnostics
Have you approved international study leave for trainees in the last 12 months?	Yes	No							
Do you actively encourage / support international study leave in your region?	Yes	Somewhat	No						
Is international study leave mandatory for progression in your training programme?	Yes	No							
In your view, does the annual NES study budget cover mandatory progression in your specialty?	Yes	Unsure	No						
If study budget does not cover all training costs in your specialty, what are the estimated personal costs to trainees on an annual basis?	No Personal Costs	Up to £1000	Up to £3000	Up to £5000	More Than £5000				
Do you feel the current £600 annual training budget is appropriate to support trainee needs and aspirations in your specialty?	No	Somewhat No	Neutral	Somewhat Yes	Yes				
Do you feel changes to NES policy not supporting international study leave is good for your training program?	No	Somewhat No	Neutral	Somewhat Yes	Yes				
With regard to international study leave, how strongly do you agree or disagree with the following?* All questions are noted in full; see Results Table 2*	Completely Agree	Somewhat Agree	Neutral	Somewhat Disagree /	Completely Disagree				
Do you have any positive comments about international study leave? What benefits has it brought trainees?	Free text response								
Do you have any negative comments about international study leave? What negatives are there in providing this opportunity to trainees in your region?	Free text response								

## References

[1] (2025). Doctors and dentists in training terms and conditions (England) 2016. https://www.nhsemployers.org/publications/doctors-and-dentists-training-terms-and-conditions-england-2016.

[2] (2025). Results of Trainees' study leave survey 2022. https://www.bgs.org.uk/resources/results-of-trainees-study-leave-survey-2022.

[3] Ercolani MG, Vohra RS, Carmichael F, Mangat K, Alderson D (2015). The lifetime cost to English students of borrowing to invest in a medical degree: a gender comparison using data from the Office for National Statistics. BMJ Open.

[4] O'Callaghan J, Mohan HM, Sharrock A, Gokani V, Fitzgerald JE, Williams AP, Harries RL (2017). Cross-sectional study of the financial cost of training to the surgical trainee in the UK and Ireland. BMJ Open.

[5] (2025). BOTA Top 5 Priorities for Trainees 2024. April.

[6] RCPsych (2024, May 2024 (2025). Access to Study Leave and Study Budget. https://www.rcpsych.ac.uk/docs/default-source/training/training/ptc/study-leave/study-leave-report-june-2024.pdf?sfvrsn=632a7c02_3.

[7] Hauss K (2020). What are the social and scientific benefits of participating at academic conferences? Insights from a survey among doctoral students and postdocs in Germany. Res Eval.

[8] Cherrstrom CA (2012). Making connections: attending professional conferences. Adult Learn.

[9] Edler J, Fier H, Grimpe C (2011). International scientist mobility and the locus of knowledge and technology transfer. Adult Learn.

[10] Smith C, Pettigrew LM, Seo HN, Dorward J (2012). Combining general practice with international work: online survey of experiences of UK GPs. JRSM Short Rep.

[11] (2025). Health Education England (HEE) Study Leave. An overview of the HEE-wide approach. https://www.hee.nhs.uk/sites/default/files/documents/Health%20Education%20England%20%28HEE%29%20Study%20Leave%20-%20An%20overview%20of%20the%20HEE-wide%20approach.pdf.

[12] (2025). Study Leave - Wales Deanery. https://heiw.nhs.wales/education-and-training/information-for-doctors-in-postgraduate-training-programmes/study-leave/.

[13] (2025). NIMDA Study Leave Policy. https://www.nimdta.gov.uk/media/pbzeqjzc/study-leave-guidelines-august-2023-v3.pdf.

[14] (2025). Study Leave - Scotland Deanery. https://www.scotlanddeanery.nhs.scot/trainee-information/study-leave/.

[15] Healthcare Employees Alliance. (2024, October 10 (2025). HCSA condemns changes to study leave budgets in Scotland. https://www.hcsa.com/news-views/news/2024/10/hcsa-condemns-changes-to-study-leave-budgets-in-scotland.aspx.

[16] Scottish Resident Doctors' Committee. (2024, October 1 (2025). SRDC position statement on changes to study leave policy. https://bmascotland.home.blog/2024/10/01/srdc-position-statement-on-changes-to-study-leave-policy/.

[17] Huh DD, Wang J, Fliotsos MJ (2022). Association between parental leave and ophthalmology resident physician performance. JAMA Ophthalmol.

[18] Castillo-Angeles M, Smink DS, Rangel EL (2021). Perspectives of US general surgery program directors on cultural and fiscal barriers to maternity leave and postpartum support during surgical training. JAMA Surg.

[19] Sharpe EE, Ku C, Malinzak EB (2021). A cross-sectional survey study of United States residency program directors' perceptions of parental leave and pregnancy among anesthesiology trainees. Can J Anaesth.

[20] Conway SE, Wang W, Prasad S (2024). Barriers to increasing paid parental leave in U.S. neurology residencies: a survey of program directors. BMC Med Educ.

[21] Magudia K, Campbell SR, Rangel EL, Arleo EK, Jagsi R, Weinstein DF, Ng TS (2021). Medical specialty board parental, caregiver, and medical leave policy updates after 2021 American Board of Medical Specialties mandate. JAMA.

[22] Chui K, Dash KK, Zaver VA, Andronic A, Allen JR, Archer JE (2024). Diversity of trauma and orthopaedic trainees and workplace culture of orthopaedic training in the United Kingdom: insights from the 2022 British Orthopaedic Trainee Association (BOTA) census. Cureus.

